# Diverging destinies: ‘social’ data within the TwinsUK cohort

**DOI:** 10.12688/wellcomeopenres.17139.1

**Published:** 2022-01-24

**Authors:** Ruth C E Bowyer, Golboo Abbasian, María Paz García, Andrew Anastasiou, Genevieve Lachance, Ellen J Thompson, Deborah Hart, Jennifer B Dowd, Claire J Steves

**Affiliations:** 1 Department of Twin Research & Genetic Epidemiology, King's College London, London, UK; 2 Leverhulme Centre for Demographic Science, Department of Sociology, University of Oxford, Oxford, Ox1 1JD, UK

**Keywords:** TwinsUK, twins, social, parenthood, working habits, sleep, sleeping habits, social support, abuse, life events, lifecourse health, spousal relationships

## Abstract

**Background**: Twins offer social scientists a unique opportunity to understand the interplay of social factors and physical and mental well-being. TwinsUK is the largest UK registry of adult mono- and dy-zygotic twins, but most of the research that utilises the cohorts’ data to date has focused on the genetic underpinnings of complex disease.

**Methods**: Following formal unstructured discussions with social scientists we identified key areas of research interest and annotated the historical data collections in TwinsUK where they could be applied to these research aims.

**Results**: We present a summary of variables identified as of key interest to researchers from the social science sphere, spanning the following domains: 1: Parenting, child rearing and pregnancies; 2: Working habits and patterns; 3: Sleeping habits and patterns; 4: Social support; 5: Negative life events; 6: Spousal relationships.

**Conclusions**: TwinsUK has a wide range of genetic and health data that would allow investigation of research questions focusing on these domains.

## Introduction

Twin studies enable researchers to disentangle and quantify the influences of relative contributions of genetic and early-life environmental factors to the variability of a trait. Twins therefore provide a unique opportunity to understand the interplay of social factors and physical and mental health and well-being, and can offer social scientists a ‘natural experiment’ where randomised controlled studies would otherwise be impossible or unethical ^1^. Using a classical MZ-DZ comparison, researchers can compare outcomes between twin-pairs who are discordant on an exposure of interest ^2^. Where there is within-pair discordance, this provides a unique tool to identify the contributing biological and environmental factors, and how these factors may alter health across the life course and diverge one twin’s health trajectory from the other (a ‘diverging destiny’).

TwinsUK is a cohort of ~14,000 volunteer adult twins. Thanks to an extensive engagement strategy and highly responsive twins, of the 69% of registered twins who have completed an in-person clinical assessment, approximately half have completed at least two follow-on visits. Consequently, extensive longitudinal data are available spanning more than 25 years in some instances. The age range of active twins is 18 – 100; the median age however is 60, meaning the cohort is particularly well-positioned for understanding the accumulation of life course exposures and age-related disease ^3^. Thus far, the TwinsUK data have been used primarily to understand the genetic basis of complex disease, leaving underutilized the extensive data capturing twins’ ‘social’ exposures as a resource to answer questions crucial to social scientists.

Here, we present a summary of the key social variables within our cohort collected prior to 2020, and the extent of twin pair discordance on each variable. Variables were identified as of key interest to researchers based on research areas in the social sciences.

## Materials and methods

### Ethical approval and consent

The data which has been generated and provided in the report has been collected via questionnaires that had received ethical approval associated with TwinsUK Biobank (19/NW/0187), Twins UK (EC04/015) or Healthy Ageing Twin Study (H.A.T.S) (07/H0802/84) studies from NHS Research Ethics Committees at the Department of Twin Research and Genetic Epidemiology, King’s College London. All members of the study hold the right to withdraw their consent and data partially or entirely upon their request. Upon joining TwinsUK, participants complete a written or online consent form to take part in subsequent research. Currently this takes place upon registration with TwinsUK, and historically this took place upon an individual’s first participation in a study or attendance of a clinic visit. TwinsUK members do not subsequently sign consent for every individual questionnaire, but they are free to choose not to complete individual questionnaires, and consent is implied when participants return their responses. TwinsUK members must be able to give consent; if the ability to consent is impaired, they are not able to join TwinsUK. TwinsUK members who wish to change their participation status with TwinsUK can do so at any time by contacting the team on email or via phone and quoting their TwinsUK participant study number. Participants are additionally able to change whether they are invited to studies and the communications they receive. TwinsUK members may also request for their data and/or samples to be destroyed at any time. Participants in questionnaires can skip questions and are not obliged to answer. Questionnaires and studies are discussed with the Twin Voluntary Advisory Panel, consisting of participants of the twin cohort, prior to implementation.

### The TwinsUK cohort

The Department of Twin Research and Genetic Epidemiology at St. Thomas’ Hospital, King’s College London (KCL), hosts the UK’s largest adult twin registry (or TwinsUK). The registry was initially set up to investigate osteoporosis and osteoarthritis, and the success of these initial studies allowed the cohort to expand, operating with rolling recruitment for new pairs aged over 18. Further details of the history of the cohort are detailed in *Verdi et al., 2019 ^3^*. The adult participants consist of 14,575 monozygotic (MZ) and dizygotic (DZ) twins aged between 18 to 100 years. Since 1992, active twins have participated in both questionnaire and clinical visits, where multiple samples and physical measures were obtained, resulting in extensive health and multiomics data (further detail of the visits is outlined in 3, 4).

### Data collection

Much of the data presented here is derived from questionnaire-based collection covering a broad range of health-related topics (e.g. co-morbidities, dietary information). Core questionnaires are collected on a rolling basis and supplemented with one-off questionnaires depending on research need. Core questionnaires are posted to individuals and returned either in person at a clinical visit or posted back. Other questionnaires (such as the first of our Covid questionnaires, as described by Suthahar *et al.* 2021 ^5^) are also posted, emailed, or collected over the phone. Where electronic questionnaires are used, Qualtrics is used to collect the data; paper questionnaire data has been processed by Abacus Data and Mailing Ltd since 2011. Some of the summaries presented below are from questionnaires that may have included eligibility criteria/been targeted at a specific subset – further details of the specific per questionnaire protocol are available from the TwinsUK data team.

### Protocol to generate this dataset

Below, we present key domains of interest to researchers seeking to understand and disentangle the genetic and environmental influence on biosocial phenotypes. Following formal unstructured discussions with social scientists we identified key areas of research interest and annotated the historical data collections in TwinsUK where they could be applied to these research aims. The domains are intended as a comprehensive, but not exhaustive, overview of pre-2020 social data within the TwinsUK cohort. A multitude of other measures are available in the cohort (see data availability) and researchers may also be interested in the longitudinal data captured by COVID-19 Personal Experience study ^5^.

For each identified domain, the relevant questions within the dataset are summarised below, including the relevant question code to expedite data requests, the question phrasing, the number of respondents per code (including ‘duplicate’ responders – where an individual may have responded to the questionnaire more than once, usually at a different date), the average and range of response year and relevant summary statistics. Some minimal data cleaning has been applied (e.g. removing responses not in possible range) but researchers should be aware that values may change slightly where they apply different filtering. All data cleaning and analysis was undertaken in R version 3.6.3 and RStudio version 1.1.423.

The strength of twins’ data is the ability to disentangle genetic and environmental influences on phenotypes. Therefore, of use to researchers in designing a study is understanding of the discordance, or number of twins who differ from their co-twin for a phenotype. We therefore present a measure of discordance as follows:

- For continuous variables, discordance between twin pairs was considered as a difference of a greater than one standard deviation of a twin from their co-twin.
- For discrete/ordinal variables, twins were considered discordant where the difference in categories was greater than one between a twin and their co-twin.
- For binary variables, twins were considered as discordant where answers were different from their co-twins.

The number of twins who had both answered the question of interest was calculated by mapping the pairs and removing any where one individual had not answered or was missing data. The reasons data could be missing from one twin are generally because the twin skipped a question, and rare cases because of a recording error or the co-twin is deceased or no longer a cohort participant. Discordance was calculated as time invariant (i.e. differing time of response between twins was not considered, although the majority of responses were recorded at similar times).

## Results

We identified six social domains within TwinsUK data pre-dating 2020, with 21 subdomains, as follows:

**Domain 1: Parenting, child rearing and pregnancies**

          1A Number of children ( Table 1A)

          1B Ever had children ( Table 1B)

          1C Age at First Pregnancy ( Table 1C)

**Domain 2: Working habits and patterns**

          2A History of Unemployment ( Table 2A)

          2B Part time work ( Table 2B)

          2C Full time care giving role and housework ( Table 2C)

          2D Working hours per week ( Table 2D)

**Domain 3: Sleeping habits and patterns**

          3A Sleep quality and patterns ( Table 3A)

          3B Trouble sleeping and insomnia ( Table 3B)

          3C Daytime sleepiness and fatigue ( Table 3C)

          3D Sleeping drugs ( Table 3D)

          3E Hours sleeping ( Table 3E)

**Domain 4: Social support**

          4A Coping with stressful events ( Table 4A)

**Domain 5: Negative life events**

          5A Major life events - past five years ( Table 5A)

          5B Major life events - past ten years ( Table 5B)

          5C Sudden death family member ( Table 5C)

          5D Ever been widowed ( Table 5D)

          5E History of abuse ( Table 5E)

**Domain 6: Spousal relationships**

          6A Current marital/relationship status ( Table 6A)

          6B Marriage history: divorce ( Table 6B)

          6C Age when first married ( Table 6C)

          6D Ever been married ( Table 6D)

          6E Length of relationships ( Table 6E)

The questions making up each domain were from questionnaires spanning between 1997 – 2018, and will reflect a mix of selected and unselected subsets of TwinsUK cohort (for detailed cohort description, see *Verdi et al., 2019 ^3^*).

### Domain 1: Parenting, child rearing and pregnancies

TwinsUK participants reported having an average of two children ( Table 1A), with a relatively stable rate of discordance across the years of questionnaire collection (approximately 55% of pairs, apart from the earliest data collection where the rate of discordance was 64.8%). Nearly three-quarters of the cohort have had children ( Table 1B) and the mean age across the measures for age of first pregnancy was 26 ( Table 1C).

**Table 1A.** Number of children. Summary of responses for TwinsUK participants in response to questions: **1A1.** (P003144) Number of children, **1A2.** (P010448) Number of children, **1A3.** (Q10_158) ‘Please write in the number of [biological children] you have had in your family’, **1A4.** (PH0000798) ‘How many pregnancies have you had resulting in a live birth?’ %F = % female responses, SD =standard deviation, %MZ = percentage of responding twin pairs who are mono-zygotic (identical) twins, N twin pairs = Number of twin pairs where both responded, %D = % of responding twin pairs considered discordant, %MZD = percentage of discordant twin pairs who are mono-zygotic.

	TwinsUK reference code	Question description	Response year (mean, range)	N responses (duplicate ids)	Population mean	SD	range	%F	Age at response (mean, SD)	N twin pairs (%MZ)	Discordant twin pairs (%D, %MZD)
1A1	P003144	Number of children	1996 (1992 – 1996)	951 (0)	1.85	1.28	0-10	99	48.7 (10.3)	472 (19.5)	306 (64.8, 17.7)
1A2	P010448	Number of children	1999	3012 (0)	1.7	1.3	0-10	93	48.8 (12.8)	1405 (28.4)	815 (58, 26.5)
			(1998 – 2001)
1A3	Q10_158	Number of biological children	2005 (all)	3437 (0)	2.25	0.91	0-10	90	55.8 (11.2)	1162 (53.4)	656 (56.5, 51.7)
1A4	PH0000798	Number of live birth	2015 (2014 – 2018)	4130 (5)	2.04	0.98	0-10	100	60.8 (12.5)	1532 (58.2)	869 (56.7, 55.5)

**Table 1B.** Ever had children. Summary of responses for TwinsUK participants in response to questions: **1B1.** (Q10_152) ‘Have you ever had any children (include biological, adopted, step-children and stillborn babies)?’ **1B2.** (Q10_157) ‘I chose not to have children’; **1B3.** (Q18_72) ‘Have you ever had a pregnancy resulting in a live birth delivery?’ **1B4.** (PH0001196) ‘Have you had a pregnancy to full term (37-42 weeks)?’ %Y= % yes response %F = % female responses, SD =standard deviation, %MZ = percentage of responding twin pairs who are mono-zygotic (identical) twins, N twin pairs = Number of twin pairs where both responded, %D = % of responding twin pairs considered discordant, %MZD = percentage of discordant twin pairs who are mono-zygotic.

	TwinsUK reference code	Question description	Response year (mean, range)	N responses (duplicate ids)	%Y	%F	Age (mean, SD)	N twin pairs (%MZ)	Discordant twin pairs (%D, %MZD)
1B1	Q10_152	Have any children	2005 (all)	4702 (0)	71.8	89.1	53.2 (13.3)	1916 (53.5)	535 (27.9, 51.0)
1B2	Q10_157	No children - personal choice	2005 (all)	1493	23.6	88	49.2 (14.8)	248 (58.9)	62 (25, 54.8)
1B3	Q18_72	Pregnancy resulting in live birth delivery	2009 (all)	3912 (0)	73.6	99.8*	55.8 (13.7)	1453 (58)	344 (23.7, 53.8)
1B4	PH0001196	Full term pregnancy	2012 (all)	3703 (0)	73.7	99.9*	58.9 (13.8)	1346 (59.1)	318 (23.6, 52.5)

**Table 1C.** Age at pregnancy of first child. Summary of responses for TwinsUK participants in response to questions: **1C1.** (P002891) Year of birth of child 1*; **1C2.** (P000942) Give in birth order, including still born and premature babies that died soon after being born, your children's year of birth (Child 1)*; **1C3.** (Q10_163) Please give, in birth order, (including stillbirth or premature babies that died soon after being born) your biological children's sex, year of birth, and if applicable, year of death. Response option: Child 1, birth*; **1C4.** (PH0001201) ‘How old were you when you had your first baby?’ %F = % female responses, SD = standard deviation, N twin pairs = Number of twin pairs where both responded, %MZ = percentage of responding twin pairs who are mono-zygotic (identical) twins, %D = % of responding twin pairs considered discordant, %MZD = percentage of discordant twin pairs who are mono-zygotic. *Age calculated as difference between birth year of child and participants birth year.

	TwinsUK reference code	Question description	Response year (mean, range)	N responses (duplicate ids)	Population mean	SD	Range	%F	Age at response (mean, SD)	N twin pairs (%MZ)	Discordant twin pairs (%D, %MZD)
1C1	P002891	Birth year child 1 ^*^	1997 (1992 - 2000)	1490 (26)	26	4.6	16 - 49	98.1	51.9 (9.5)	595 (36)	197 (33.1, 31)
1C2	P000942	Birth order child 1 year born ^*^	1998 (1996 - 2001)	3793 (386)	25.9	4.7	14 - 46	95	50.8 (10.5)	1420 (30)	404 (28.5, 27.7)
1C3	Q10_163	Children birth order child 1 year born ^*^	2005 (all)	3466 (0)	26.8	5	16 - 50	90	55.9 (11.1)	1182 (52.5)	395 (33.4, 46.6)
1C4	PH0001201	Age at first pregnancy	2012 (all)	1625 (0)	25.3	4.6	16 - 43	100	62 (11.3)	343 (57.4)	97 (28.3, 50.5)

### Domain 2: Working habits and patterns

The average number of years spent without employment within the cohort was 9.9 ( Table 2A); with the average length of years spent working in part time work being 11.1 years ( Table 2B). Nearly half of respondents (45.2%) had worked in a fulltime care giving role ( Table 2C). The average working hours per week reported was 31.7, but this was highly variable (SD=12.9, Table 2D).

**Table 2A.** History of unemployment. Summary of responses for TwinsUK participants in response to question: **2A1.** (Q11A_44) ‘In your life, since turning 16 years old, approx. how many years altogether have you spent working without employment?’ %F = % female responses, SD = standard deviation, N twin pairs = Number of twin pairs where both responded, %MZ = percentage of responding twin pairs who are mono-zygotic (identical) twins, %D = % of responding twin pairs considered discordant, %MZD = percentage of discordant twin pairs who are mono-zygotic.

	TwinsUK reference code	Question description	Response year (mean, range)	N responses (duplicate ids)	Population mean	SD	Range	%F	Age at response (mean, SD)	N twin pairs (%MZ)	Discordant twin pairs (%D, %MZD)
2A1	Q11A_44	Without employment - total years since age 16	2008 (2004 – 2014)	3633 (0)	9.9	10.2	0–75	90.7	54.5 (15)	1326 (59)	313 (23.6, 54.6)

**Table 2B.** Part time work. Summary of responses for TwinsUK participants in response to question: **2B1.** (Q11A_43) ‘In your life, since turning 16 years old, approx. how many years altogether have you spent working in part-time employment?’ %F = % female responses, SD = standard deviation, N twin pairs = Number of twin pairs where both responded, %MZ = percentage of responding twin pairs who are mono-zygotic (identical) twins, %D = % of responding twin pairs considered discordant, %MZD = percentage of discordant twin pairs who are mono-zygotic.

	TwinsUK reference code	Question description	Response year (mean, range)	N responses (duplicate ids)	Population mean	SD	Range	%F	Age at response (mean, SD)	N twin pairs (%MZ)	Discordant twin pairs (%D, %MZD)
2B1	Q11A_43	Working part time employment - total years since age 16	2008 (2004 – 2014)	4756 (935)	11.1	10	0–60	92.7	54.2 (14.3)	1414 (57)	426 (30.1, 48.8)

**Table 2Ci.** Full time care giving role and housework. i. Summary of responses for TwinsUK participants in response to question: **2C1.** (Q11A_72) Have you ever worked in the following areas and/or capacities: housewife / full-time parent / carer? %F = % female responses, %Yes = % yes response, N twin pairs = Number of twin pairs where both responded, %MZ = percentage of responding twin pairs who are mono-zygotic (identical) twins, %D = % of responding twin pairs considered discordant, %MZD = percentage of discordant twin pairs who are mono-zygotic.

	TwinsUK reference code	Question description	Response year (mean, range)	N responses (duplicate ids)	%Yes	%F	Age (mean, SD)	N twin pairs (%MZ)	Discordant twin pairs (%D, %MZD)
2C1	Q11A_72	Work area housewife parent carer	2008 (2004 – 2014)	7084 (1672)	45.2	88.3	54.2 (14.3)	2583 (59)	780 (30.2, 51.7)

**Table 2Cii.** Full time care giving role and housework ii. Summary of responses for TwinsUK participants in response to question: **2C2.** (Q17D_28) During the last week, how many hours did you spend on each of the following physical activities: Housework/Childcare/Carer? %F = % female responses, SD =standard deviation, N twin pairs = Number of twin pairs where both responded, %MZ = percentage of responding twin pairs who are mono-zygotic (identical) twins, %D = % of responding twin pairs considered discordant, %MZD = percentage of discordant twin pairs who are mono-zygotic.

	TwinsUK reference code	Question description	Response year (mean, range)	N responses (duplicate ids)	Population mean	SD	Range	%F	Age at response (mean, SD)	N twin pairs (%MZ)	Discordant twin pairs (%D, %MZD)
2C2	Q17D_28	Physical activities hours last week housework childcare carer	2008 (2007 – 2010)	3408 (2)	2.3	0.8	0 – 3	99.5	58.4 (10.2)	1600 (51.8)	813 (50.8, 50.3)

**Table 2D.** Working hours per week. Summary of responses for TwinsUK participants in response to question: **2D1** (Q2_289) How many hours per week do you usually work in your job/business? Please exclude meal breaks and paid overtime. %F = % female responses, SD = standard deviation, N twin pairs = Number of twin pairs where both responded, %MZ = percentage of responding twin pairs who are mono-zygotic (identical) twins, %D = % of responding twin pairs considered discordant, %MZD = percentage of discordant twin pairs who are mono-zygotic.

	TwinsUK reference code	Question description	Response year (mean, range)	N responses (duplicate ids)	Population mean	SD	Range	%F	Age at response (mean, SD)	N twin pairs (%MZ)	Discordant twin pairs (%MZD)
2D1	Q2_289	Work hours per week	1999 (1999– 2000)	3857 (0)	31.7	12.9	0 – 99	91.1	44.4 (11)	1266 (50.2)	470 (37.1, 46.2)

### Domain 3: Sleeping habits and patterns

Over 1,000 twin pairs were discordant for their sleep quality at time of response ( Table 3Ai) although many (75.5%) reported they had a regular sleeping pattern during the working week ( Table 3Aii). Most participants reported slight levels of insomnia ( Table 3B) and daytime sleepiness and fatigue ( Table 3C). 15.5% and 6.6% of responders reported use of non-prescription and prescription sleeping medications respectively ( Table 3D). The average hours slept per night was between 7–8 hours ( Table 3E).

**Table 3Ai.** Sleep quality and patterns i. Summary of responses for TwinsUK participants in response to question: How would you describe your sleep quality over the last month? With the following response options: 1. Very poor, 2. Poor, 3. Satisfactory, 4. Good, 5. Very good. %F = % female responses, SD = standard deviation, N twin pairs = Number of twin pairs where both responded, %MZ = percentage of responding twin pairs who are mono-zygotic (identical) twins, %D = % of responding twin pairs considered discordant, %MZD = percentage of discordant twin pairs who are mono-zygotic.

	TwinsUK reference code	Question description	Response year (mean, range)	N responses (duplicate ids)	Population median	Range	%F	Age at response (mean, SD)	N twin pairs (%MZ)	Discordant twin pairs (%D, %MZD)
3A1	PH0000362	Sleep quality last month	2014 (2014 – 2018)	6228 (8)	3	1 – 5	87.36	57 (15.7)	2689 (61.8)	1691 (62.9, 59.1)

**Table 3Aii.** Sleep quality and patterns ii. Summary of responses for TwinsUK participants in response to question: Do you have a regular sleep pattern during the working week? %F = % female responses, %Yes = % yes response, N twin pairs = Number of twin pairs where both responded, %MZ = percentage of responding twin pairs who are mono-zygotic (identical) twins, %D = % of responding twin pairs considered discordant, %MZD = percentage of discordant twin pairs who are mono-zygotic.

	TwinsUK reference code	Question description	Average response year (range)	N responses (duplicate ids)	%Yes	%F	Age at response (mean, SD)	N twin pairs (%MZ)	Discordant twin pairs (%D, %MZD)
3A2	PH0001314	Regular sleeping pattern	2013 (all)	3796 (7)	75.5	89.6	59.5 (13.8)	1305 (60)	377 (28.9, 53.3)

**Table 3B.** Trouble sleeping and insomnia. Summary of responses for TwinsUK participants in response to question: **3B1.** (P001470) ‘Have you recently had difficulty in staying asleep once you are off?’ With the following response options: 0: Not at all, 1: No more than usual, 2: Rather more than usual, 3: Much more than usual **3B2**. (P001469) ‘Have you recently lost much sleep over worry?’ With the following response options: 0: Not at all, 1: No more than usual, 2: Rather more than usual, 3: Much more than usual; **3B3.** (Q4_23) ‘How often did you have insomnia (trouble sleeping) in the last year?’ With the following response options: 0: Does not occur or less than once a month, 1: Occurs about once a month, 2: Occurs about once a week, 3: Occurs several times a week, 4: Occurs daily; **3B4.** (Q4_24) ‘How much did the insomnia (trouble sleeping) bother you in the last year?’ With the following response options: 0: Not a problem, 1: It bothers me slightly, 2: It bothers me moderately, 3: It bothers me severely, 4: It bothers me extremely. %F = % female responses, SD = standard deviation, N twin pairs = Number of twin pairs where both responded, %MZ = percentage of responding twin pairs who are mono-zygotic (identical) twins, %D = % of responding twin pairs considered discordant, %MZD = percentage of discordant twin pairs who are mono-zygotic.

	TwinsUK reference code	Question description	Average response year (range)	N responses (duplicate ids)	Population median	Range	%F	Age at response (mean, SD)	N twin pairs (%MZ)	Discordant twin pairs (%D, %MZD)
3B1	P001470	Recently had difficulty staying asleep	1997 (1992 - 2004)	6430 (1914)	1	0 – 3	97	50 (12.7)	2209 (35)	1192 (54, 29.4)
3B2	P001469	GHQ Recently lost sleep over worry	1997 (1992 - 2004)	6430 (1915)	1	0 – 3	97	50 (12.7)	2208 (35)	1137 (51.5, 30.1)
3B3	Q4_23	Insomnia frequency last year	2000 (all)	4919 (0)	1	0 – 4	90	49.6 (13)	1901 (49.8)	1159 (61, 47.2)
3B4	Q4_24	Insomnia bothering you last year	2000 (all)	4847(0)	1	0 – 4	90.2	49.6 (13)	1849 (50)	1130 (61.1, 47.7)

**Table 3C.** Daytime sleepiness and fatigue. Summary of responses for TwinsUK participants in response to question: **3C1.** (Q4_25) ‘How often did you get fatigue (tiredness) in the last year?’ With the following response options: 0: Does not occur or less than once a month, 1: Occurs about once a month, 2: Occurs about once a week, 3: Occurs several times a week, 4: Occurs daily; **3C2.** (Q4_26) ‘How much did fatigue (tiredness) bother you in the last year?’ 0: Not a problem, 1: It bothers me slightly, 2: It bothers me moderately, 3: It bothers me severely, 4: It bothers me extremely; **3C3.** (Q2_74) ‘How often, if at all, do you experience sleepiness during the daytime?’ With the following responses: 1: Never, 2: Rarely, 3: Sometimes, 4: Frequently, 5: Always; **3C4**. (Q11A_159) ‘How often, if at all, do you experience sleepiness in the daytime?’ With the following responses: 1: Never, 2: Rarely, 3: Sometimes, 4: Frequently, 5: Always. %F = % female responses, SD = standard deviation, N twin pairs = Number of twin pairs where both responded, %MZ = percentage of responding twin pairs who are mono-zygotic (identical) twins, %D = % of responding twin pairs considered discordant, %MZD = percentage of discordant twin pairs who are mono-zygotic.

	TwinsUK reference code	Question description	Response year (mean, range)	N responses (duplicate ids)	Population median	Range	%F	Age at response (mean, SD)	N twin pairs (%MZ)	Discordant twin pairs (%D, %MZD)
3C1	Q4_25	Fatigue frequency last year	2000 (all)	4931 (0)	2	0 – 4	90	49.6 (13)	1906 (49.9)	1321 (69.3, 47.3)
3C2	Q4_26	Fatigue bothering you last year	2000 (all)	4931 (0)	1	0 – 4	90	49 (13)	1864 (49.9)	1167 (62.6, 48.2)
3C3	Q2_74	Daytime sleepiness	1999 (1999 – 2000)	5988 (0)	3	1 – 5	91.8	48.6 (13.1)	2553 (49.5)	1484 (58.1, 45)
3C4	Q11A_159	Experiencing daytime sleepiness	2008 (2004 – 2014)	7063 (1664)	3	1 – 5	88.3	54.2 (14.3)	2576 (59.1)	1464 (56.8, 55.4)

**Table 3D.** Use of sleeping drugs. Summary of responses for TwinsUK participants in response to question: **3D1** (Q11B_207) ‘Have you ever taken this drug: Sleeping tablets?; **3D2** (Q11B_30) ‘Have you ever taken this drug every day for over a month: Sleeping tablets / tranquilisers?’ %F = % female responses, %Yes = % yes response, SD = standard deviation, N twin pairs = Number of twin pairs where both responded, %MZ = percentage of responding twin pairs who are mono-zygotic (identical) twins, %D = % of responding twin pairs considered discordant, %MZD = percentage of discordant twin pairs who are mono-zygotic.

	TwinsUK reference code	Question description	Response year (mean, range)	N responses (duplicate ids)	%Yes	%F	Age at response (mean ,SD)	N twin pairs (%MZ)	Discordant twin pairs (%D, %MZD)
3D1	Q11B_207	Non-prescription drugs sleeping tablets	2007 (2004–2014)	7045 (3609)	15.5	88	51.7 (14.5)	2401 (60.8)	532 (22.2, 60.7)
3D2	Q11B_30	Prescription drugs sleeping tranquilisers daily month	2007 (2004–2014)	7045 (1763)	6.6	88	51.7 (14.4)	2401 (60.8)	241 (10, 62.2)

**Table 3E.** Hours sleeping. Summary of responses for TwinsUK participants in response to question: **3E1** (Q7_16) ‘On average, how many hours per night do you sleep during a working week (Sun-Thru night)?’ with the following possible responses: 1: Less than 6 hours, 2: 6–7 hours, 3: 7–8 hours, 4: 8–9 hours, 5: More than 9 hours, **3E2** (Q7_17) ‘On average, how many hours per night do you sleep during the weekend (Fri-Sat night)?’ with the following possible responses: 1: Less than 6 hours, 2: 6–7 hours, 3: 7–8 hours, 4: 8–9 hours, 5: More than 9 hours, **3E3** (Q7_18) ‘On average, how many hours per night do you sleep during a holiday?’ with the following possible responses: 1: Less than 6 hours, 2: 6–7 hours, 3: 7–8 hours, 4: 8–9 hours, 5: More than 9 hours, **3E4** (Q19_29) ‘On average how many hours do you sleep each night during a week (Monday-Thursday)? Please use decimals to indicate less than a whole number, e.g. 7.5.’ (continuous responses) **3E5** (Q19_30) ‘On average how many hours do you sleep each night at the weekend (Friday-Saturday)? Please use decimals to indicate less than a whole number, e.g. 7.5.’ (continuous responses). **3E6** (PH0000360) ‘About how many hours sleep do you get in every 24 hours during the working week? (hrs)’ (continuous responses). %F = % female responses, SD = standard deviation, N twin pairs = Number of twin pairs where both responded, %MZ = percentage of responding twin pairs who are mono-zygotic (identical) twins, %D = % of responding twin pairs considered discordant, %MZD = percentage of discordant twin pairs who are mono-zygotic.

	TwinsUK reference code	Question description	Response year (mean, range)	N responses (duplicate ids)	Population median	SD	Range	%F	Age at response (mean, SD)	N twin pairs (%MZ)	Discordant twin pairs (%D, %MZD)
3E1	Q7_16	Sleep during working week hours per night	2002 (all)	5121 (9)	2	0.9	1 – 5	88.6	51 (13)	2039 (49.9)	1227 (60.1, 47.4)
3E2	Q7_17	Sleep during weekend hours per night	2002 (all)	5014 (9)	3	1	1 – 5	88.7	50.9 (12.9)	1955 (50.1)	377 (19.3, 45.1)
3E3	Q7_18	Sleep during holiday hours per night	2002 (all)	4932 (9)	3	1	1 – 5	88.6	50.9 (13)	1897 (50)	376 (19.8, 48.1)
3E4	Q19_29	Sleep weekday hours each night	2010 (all)	2827 (0)	7	1.3	0 – 39	88	52.5 (13.6)	855 (65.8)	217 (25.4, 56.7)
3E5	Q19_30	Sleep weekend hours each night	2010 (all)	2828 (0)	7.5	1.36	0.21 – 21	88	52.5 (13.6)	856 (65.8)	271 (31.7, 59.7)
3E6	PH0000360	Hours slept during working week day	2014 (2014 - 2018)	5970 (8) *	7	1.14	1 – 2	87.37	56.9 (15.8)	2499 (62.1)	495 (19.8, 51.7)

*2 individuals removed for stating greater than 24 hours

### Domain 4: Social support

Over 60% of responding twin pairs were discordant for their ability to get emotional support from others and handling difficult situations without needing emotional support from others ( Table 4A).

**Table 4A.** Social support. Summary of responses for TwinsUK participants in response to question: **4A1.** (Q9_144) ‘Please indicate what you generally do and feel when you experience stressful events. - I get emotional support from others.’ With possible responses: 1: I usually don't do this at all, 2: I usually do this a little bit, 3: I usually do this a medium amount, 4: I usually do this a lot, **4A2.** (Q16_145) ‘Please read each statement and tick the box that best reflects your degree of agreement or disagreement with that statement - I can handle difficult situations without needing emotional support from anyone else.’ With possibly responses: 1: Strongly Agree, 2: Agree, 3: Neutral (neither agree or disagree), 4: Disagree, 5: Strongly Disagree. %Yes = % yes response, %F = % female responses, SD = standard deviation, %MZ = percentage of responding twin pairs who are mono-zygotic (identical) twins, N twin pairs = Number of twin pairs where both responded, %D = % of responding twin pairs considered discordant, %MZD = percentage of discordant twin pairs who are mono-zygotic.

	TwinsUK reference code	Question description	Response year (mean, range)	N responses (duplicate ids)	Population median	Range	%F	Age at response (mean, SD)	N twin pairs (%MZ)	Discordant twin pairs (%D, %MZD)
4A1	Q9_144	Coping with stressful events - emotional support from others	2004 (all)	5468 (2)	3	1 -4	89	52.7 (13)	2285 (51.4)	1460 (63.8, 49.5)
4A2	Q16_145	Handling difficult situations without emotional support	2008 (all)	4028 (0)	3	1 - 5	89.4	56.1 (13.1)	1530 (56.7)	966 (63, 54.97)

### Domain 5: Negative life events

Over half (54.9%) of responders reported that they had experienced a self-defined major negative life event related to their home and family in the five years prior to questionnaire ( Table 5Ai); events related to home and family were also the most stressful ( Table 5Aii). Comparatively lower rates of major life events were reported in ten years prior to questionnaire ( Table 5Bi) but with similar stress levels ( Table 5Bii). Fathers were more likely to have been reported as dying suddenly (13.5%) than other family members ( Table 5C). In 2013, 22.9% of responders reported being widowed; of the 398 twin pairs responding 26% (n=104) were discordant ( Table 5D). Reports of childhood abuse was 6.3-6.7% with within pair discordance ranging between 9.7-12% of responding pairs ( Table 5E).

**Table 5Ai.** Major life events – past 5 years. Summary of responses for TwinsUK participants in response to question: Please indicate whether you have experienced any major events in any of the following areas of your life during the past 5 years (2000-2005): **5A1.**(Q10_33) Major events with your Health, **5A2.** (Q10_37) Major events in your Home and Family, **5A3.** (Q10_41) Major events with your Work, **5A4.** (Q10_45) Major events in your Interpersonal Life, **5A5.**(Q10_49) Major Economic or Legal event. %Yes = % yes response, %F = % female responses, SD = standard deviation, N twin pairs = Number of twin pairs where both responded, %MZ = percentage of responding twin pairs who are mono-zygotic (identical) twins, %D = % of responding twin pairs considered discordant, %MZD = percentage of discordant twin pairs who are mono-zygotic.

	TwinsUK reference code	Question description	Response year (mean, range)	N responses (duplicate ids)	%Yes	%F	Age (mean, SD)	N twin pairs (%MZ)	Discordant twin pairs (%D, %MZD)
5A1	Q10_33	Major life events (past 5yrs) - health	2005(all)	4677 (0)	36	89.3	52.6 (13.4)	1855 (53.9)	1091 (58.8, 51)
5A2	Q10_37	Major life events (past 5yrs) – home & family	2005(all)	4681 (0)	54.9	89.2	52.6 (13.4)	1893 (54.1)	1007 (53.2, 52.03)
5A3	Q10_41	Major life events (past 5yrs) – work	2005(all)	4551 (0)	36.2	89.2	52.1(13.2)	1794 (54.3)	944 (52.6, 52)
5A4	Q10_45	Major life events (past 5yrs) - interpersonal	2005(all)	4596 (0)	24.9	89.3	52.5 (13.4)	1832 (53.7)	932 (50.9, 51.4)
5A5	Q10_49	Major life events (past 5yrs) - economic & legal	2005(all)	4665 (0)	19.5	89.2	52.5 (13.3)	1881 (53.6)	911 (48.4, 52.3)

**Table 5Aii.** Major life events – past 5 years ii (stress level). Summary of responses for TwinsUK participants in response to question: For each event that you experienced [as in 5Ai] please rate how stressful it was on a scale 0 to 10 where 0 = “not at all stressful” and 10 = “extremely stressful”. **5A6.** (Q10_34) Major events with your Health **5A7.** (Q10_38) Major events in your Home and Family **5A8.** (Q10_42) Major events with your Work, **5A9.** (Q10_46) Major events in your Interpersonal Life, %F = % female responses, SD = standard deviation, N twin pairs = Number of twin pairs where both responded, %MZ = percentage of responding twin pairs who are mono-zygotic (identical) twins, %D = % of responding twin pairs considered discordant. Discordance and twin pairs consider all responders to 5Ai, not just affirmative responses, %MZD = percentage of discordant twin pairs who are mono-zygotic.

	TwinsUK reference code	Question description	Response year (mean, range)	N responses (duplicate ids)	Population median	Range	%F	Age (mean, SD)	N twin pairs (%MZ)	Discordant twin pairs (%D, %MZD)
5A6	Q10_34	Major life events (past 5yrs) - health - stress level	2005(all)	4677 (0)	7	0-10	89.3	52.6 (13.4)	1855 (53.9)	217 (11.7, 51.6)
5A7	Q10_38	Major life events (past 5yrs) -home & family - stress level	2005(all)	4681 (0)	8	0-10	89.2	52.6 (13.4)	1893 (54.1)	187 (9.9, 49.7)
5A8	Q10_42	Major life events (past 5yrs) -work - stress level	2005(all)	4551 (0)	6	0-10	89.2	52.1 (13.2)	1794 (54.3)	222 (12.4, 50.9)
5A9	Q10_46	Major life events (past 5yrs) - interpersonal - stress level	2005(all)	4596 (0)	6	0-10	89.3	52.46 (13.4)	1832 (53.7)	170 (9.3, 51.2)
5A10	Q10_50	Major life events (past 5yrs) – economic & legal - stress level	2005(all)	4665 (0)	5	0-10	89.2	52.5 (13.3)	1881 (53.6)	139 (7.4, 50.4)

**Table 5Bi.** Major life events – past 10 years i. Summary of responses for TwinsUK participants in response to question: Please indicate whether you have experienced any major events in any of the following areas of your life between 5 and 10 years ago (1995-1999): **5B1.**(Q10_33) Major events with your Health, **5B2.** (Q10_37) Major events in your Home and Family, **5B3.** (Q10_41) Major events with your Work, **5B4.** (Q10_45) Major events in your Interpersonal Life, **5B5.** (Q10_49) Major Economic or Legal event. %Yes = % yes response, %F = % female responses, SD = standard deviation, N twin pairs = Number of twin pairs where both responded, %MZ = percentage of responding twin pairs who are mono-zygotic (identical) twins, %D = % of responding twin pairs considered discordant, %MZD = percentage of discordant twin pairs who are mono-zygotic.

	TwinsUK reference code	Question description	Response year (mean, range)	N responses (duplicate ids)	%Yes	%F	Age (mean, SD)	N twin pairs (%MZ)	Discordant twin pairs (%D, %MZD)
5B1	Q10_33	Major life events (past 10yrs) - health	2005	4659 (0)	25.9	89.2	52.6 (13.4)	1875 (54.1)	1002 (53.4, 50.5)
5B2	Q10_37	Major life events (past 10yrs) – home & family	2005	4648 (0)	43.7	89.4	52.6 (13.4)	1869 (54.3)	1064 (56.9, 51.6)
5B3	Q10_41	Major life events (past 10yrs) – work	2005	4502 (0)	27.4	89.2	52.2 (13.2)	1758 (54.4)	940 (53.5, 53.30)
5B4	Q10_45	Major life events (past 10yrs) - interpersonal	2005	4551 (0)	20.5	89.2	52.5 (13.4)	1797 (54.2)	874 (48.6, 53)
5B5	Q10_49	Major life events (past 10yrs) - economic & legal	2005	4612 (0)	14	89.3	52.5 (13.4)	1838 (54.1)	784 (42.7, 51.9)

**Table 5Bii.** Major life events – past 10 years ii (stress level). Summary of responses for TwinsUK participants in response to question: For each event that you experienced [as in 5Bi] please rate how stressful it was on a scale 0 to 10 where 0 = “not at all stressful” and 10 = “extremely stressful”. **5B6.** (Q10_34) Major events with your Health, **5B7.** (Q10_38) Major events in your Home and Family, **5B8.** (Q10_42) Major events with your Work, **5B9** (Q10_46) Major events in your Interpersonal Life, %F = % female responses, SD = standard deviation, N twin pairs = Number of twin pairs where both responded, %MZ = percentage of responding twin pairs who are mono-zygotic (identical) twins, %D = % of responding twin pairs considered discordant, %MZD = percentage of discordant twin pairs who are mono-zygotic.

	TwinsUK reference code	Question description	Response year (mean, range)	N responses (duplicate ids)	Population median	Range	%F	Age (mean, SD)	N twin pairs (%MZ)	Discordant twin pairs (%D, %MZD)
5B6	Q10_34	Major life events (past 10yrs) - health - stress level	2005(all)	4659 (0)	6	0-10	89.2	52.6 (13.4)	1875 (54.1)	184 (9.8, 51.6)
5B7	Q10_38	Major life events (past 10yrs) -home & family - stress level	2005(all)	4648 (0)	7	0-10	89.4	52.6 (13.4)	1869 (54.3)	277 (14.8, 52.4)
5B8	Q10_42	Major life events (past 10yrs) -work - stress level	2005(all)	4502 (0)	5	0-10	89.2	52.2 (13.2)	1758 (54.4)	158 (9, 45.6)
5B9	Q10_46	Major life events (past 10yrs) - interpersonal - stress level	2005(all)	4551 (0)	5	0-10	89.2	52.5 (13.4)	1797 (54.2)	277 (48, 15.4)
5B10	Q10_50	Major life events (past 10yrs) – economic & legal - stress level	2005(all)	46610 (0)	5	0-10	89.2	52.5 (13.3)	1881 (53.6)	139 (50.4)

**Table 5C.** Sudden death of a family member. Summary of responses for TwinsUK participants in response to question: **5C1**. (P003093) Is there anyone in your family who died suddenly? – response option: Mother. **5C2.** (P003094) Is there anyone in your family who died suddenly? – response option: Father. **5C3**. (P003095) Is there anyone in your family who died suddenly? – response option: any sister. **5C4.** (P003096) Is there anyone in your family who died suddenly? – response option: any brother. **5C5.** (P003097) Is there anyone in your family who died suddenly? – response option: any child. %Yes = % yes response, %F = % female responses, SD = standard deviation, N twin pairs = Number of twin pairs where both responded, %MZ = percentage of responding twin pairs who are mono-zygotic (identical) twins, %D = % of responding twin pairs considered discordant, %MZD = percentage of discordant twin pairs who are mono-zygotic.

	TwinsUK reference code	Question description	Response year (mean, range)	N responses (duplicate ids)	%Yes	%F	Age (mean, SD)	N twin pairs (%MZ)	Discordant twin pairs (%D, %MZD)
5C1	P003093	Mother died suddenly	1999 (1997-2001)	3638 (148)	7.31	93.8	47.5 (12.9)	1782 (28.5)	0
5C2	P003094	Father died suddenly	1999 (1997-2001)	3628 (140)	13.5	93.8	47.6 (12.9)	1779 (28.6)	0
5C3	P003095	Sister died suddenly	1999 (1997-2001)	1909 (76)	2.7	92.6	47.4 (12.6)	933 (27)	0
5C4	P003096	Brother died suddenly	1999 (1997-2001)	2041 (68)	5.6	94.4	47.4 (12.5)	1001 (26.9)	0
5C5	P003097	Child died suddenly	1999 (1997-2001)	2597 (120)	1.1	94.4	51 (10.5)	1063 (27.1)	21 (2, 23.8)

**Table 5D.** Ever been widowed. Summary of responses for TwinsUK participants in response to question: **5D1.** (Q11A_35) Have you ever been widowed? **5D2**. (Q17D_10) In the past ten years were you widowed? **5C3**. (PH0001333) What is your current relationship status? -response option: widowed. %Yes = % yes response, %F = % female responses, SD = standard deviation, , N twin pairs = Number of twin pairs where both responded, %MZ = percentage of responding twin pairs who are mono-zygotic (identical) twins, %D = % of responding twin pairs considered discordant, %MZD = percentage of discordant twin pairs who are mono-zygotic.

	TwinsUK reference code	Question description	Response year (mean, range)	N responses (duplicate ids)	%Yes	%F	Age (mean, SD)	N twin pairs (%MZ)	Discordant twin pairs (%D, %MZD)
5D1	Q11A_35	Ever been widowed	2008 (2004 - 2014)	5635 (1340)	9.6	88.9	57.2 (11.5)	1921 (56.2)	223 (11.6, 52.5)
5D2	Q17D_10	Widowed in past ten years	2008 (2007 - 2010)	710 (0)	25.4	99.2	56.6 (11.8)	90 (60)	22 (24.4, 68.2)
5D3	PH0001333	Relationship status widowed	2013 (all)	1531 (8)	22.9	92.7	66.9 (11.4)	398 (54.8)	104 (26.1, 47.1)

**Table 5E.** History of abuse. Summary of responses for TwinsUK participants in response to question: **5E1.** (Q10_227) As a child, were you ever a victim of physical or sexual abuse? (As this may be linked to some medical conditions.) - response option: physical abuse **5E2**. (Q10_228) As a child, were you ever a victim of physical or sexual abuse? (As this may be linked to some medical conditions.) - response option: sexual abuse %Yes = % yes response, %F = % female responses, SD = standard deviation, N twin pairs = Number of twin pairs where both responded, %MZ = percentage of responding twin pairs who are mono-zygotic (identical) twins, %D = % of responding twin pairs considered discordant, %MZD = percentage of discordant twin pairs who are mono-zygotic.

	TwinsUK reference code	Question description	Response year (mean, range)	N responses (duplicate ids)	%Yes	%F	Age (mean, SD)	N twin pairs (%MZ)	Discordant twin pairs (%D, %MZD)
5E1	Q10_227	Physical abuse victim child	2005	4594 (0)	6.7	89	52.6 (13.5)	1839 (55)	179 (9.7, 51.4)
5E2	Q10_228	Sexual abuse victim child	2005	4595 (0)	6.3	89.3	52.5 (13.4)	1836 (53.9)	222 (12, 48.7)

### Domain 6: Spousal relationships

The majority (72-76%) of participants reported being married or in a relationship at time of questionnaire ( Table 6Ai– Table 6Aii), with divorce rates between 20-36.2% of responders depending on the year/questionnaire ( Table 6Bi), and the mean number of times married was 1.2 ( Table 6Bii). The mean age of first marriage was 24.3 years with a 22.8% rate of discordance ( Table 6C). 88.9% responders reported that they had ever been married ( Table 6D) with the average length of relationship with current partner 30 years ( Table 6E).

**Table 6Ai.** Current Marital/Relationship status i. Summary of responses for TwinsUK participants in response to question: **6A1.** (Q11A_30) Are you currently married?, **6A2.** (Q11A_31) Are you currently separated from your spouse although not divorced? **6A3.** (Q14_4) Are you currently in a relationship?, **6A4** (Q17D_5) Are you currently in a relationship?, **6A5** (Q17D_7) Is your relationship status different from ten years ago?, **6A6** (PH0001326) What is your current relationship status? – response option: Single, not seeing anyone. **6A7** (PH0001327) What is your current relationship status? – response option: Single, but dating someone. **6A8.** (PH0001330) What is your current relationship status? – response option: Married. **6A9** (PH0001331) What is your current relationship status? – response option: Separated. **6A10 (**PH0001332) What is your current relationship status? – response option: Divorced. **6A11.** (PH0001334) Are you currently married or in a relationship? %Yes = % yes response, %F = % female responses, SD = standard deviation, N twin pairs = Number of twin pairs where both responded, %MZ = percentage of responding twin pairs who are mono-zygotic (identical) twins, %D = % of responding twin pairs considered discordant, %MZD = percentage of discordant twin pairs who are mono-zygotic.

	TwinsUK reference code	Question description	Response year (mean, range)	N responses (duplicate ids)	%Yes	%F	Age (mean, SD)	N twin pairs (%MZ)	Discordant twin pairs (%D, %MZD)
6A1	Q11A_30	Currently married	2008 (2004- 2014)	5695 (2690)	76.7	88.9	57.1 (11.6)	1866 (57)	604 (32.4, 56.1)
6A2	Q11A_31	Currently separated from spouse	2009 (2004- 2014)	4241 (1611)	4	87.7	56.4 (11.3)	1223 (56.1)	97 (7.9, 56.1)
6A3	Q14_4	Currently in relationship	2008	1856 (0)	43.5	88.2	52.2 (16.1)	361 (62.1)	120 (33.2, 58.3)
6A4	Q17D_5	Currently in a relationship	2009 (2007- 2010)	1304 (0)	38.8	99.4	58.21 (11.9)	280 (57.1)	81 (28.9, 46.9)
6A5	Q17D_7	Relationship status compared to 10 years ago	2009 (2007- 2010)	3422 (4)	20.3	99.6	58.3 (10.1)	1614 (52.3)	456 (29.3, 52.9)
6A6	PH0001326	Relationship status single dating someone	2013	2777 (0)	18.9	88.5	56.60 (13.7)	809 (65.8)	144 (17.8, 59.7)
6A7	PH0001327	Relationship status single not dating someone	2013	2590 (0)	2.2	88.4	56 (13.6)	752 (66.2)	25 (3.3, 72)
6A8	PH0001330	Relationship status married	2013	3384 (0)	72.1	89.3	59.6 (13)	1074 (62.2)	309 (28.8, 59.2)
6A9	PH0001331	Relationship status separated	2013	2588 (0)	1.9	88.4	56.1 (13.6)	748 (66.3)	19 (2.5, 57.9)
6A10	PH0001332	Relationship status divorced	2013	2697 (0)	12	88.6	56.6 (13.6)	772 (66)	123 (15.9, 58.5)
6A11	PH0001334	Relationship status	2013	3868 (0)	74	89.7	61.8 (12.8)	1367 (59.5)	411 (54.7)

**Table 6Aii.** Current Marital/Relationship status ii. Summary of responses for TwinsUK participants in response to question: **6A12** (Q2_70) ‘What is your marital status?’, with possible responses: Single (1), Co-habiting (2), Married (3), Divorced/separated (4), **6A13** (Q6_3) ‘What is your current marital status?’, with possible responses: Single (1), Married (and living with husband) (2), Living with a partner who is male (3), Living with a partner who is female (4), Divorced/separated (5), Widowed (6), **6A14** (Q12_3) ‘What is your current marital status?’, with possible responses: Single (never married) (1), Married (2), Living with a significant other (male partner) (3), Living with a significant other (female partner) (4), In a relationship (with male partner), but not living together (5), In a relationship (with female partner), but not living together (6), Divorced / separated (and now single) (7), Widow (and now single) (8), **6A15** (Q14_3) ‘What is your current marital status?’, with possible responses: Married (1), Single (whether in a relationship or not) (2), Divorced (whether in a relationship or not) (3), Separated (whether in a relationship or not) (4), Widowed (whether in a relationship or not) (5), **6A16** (Q17D_4) ‘What is your current marital status?’, with possible responses: Married (and living together) (1), Married (but living apart) (2), Single (whether in a relationship or not) (3), Divorced (4), Separated (5), Widowed (6). %F = % female responses, SD = standard deviation, N twin pairs = Number of twin pairs where both responded, %MZ = percentage of responding twin pairs who are mono-zygotic (identical) twins, %D = % of responding twin pairs considered discordant, %MZD = percentage of discordant twin pairs who are mono-zygotic.

	TwinsUK reference code	Question description	Response year (mean, range)	N responses (duplicate ids)	Population mode	Range	%F	Age at response (mean, SD)	N twin pairs (%MZ)	Discordant twin pairs (%D, %MZD)
6A12	Q2_70	Marital status	1999 (1999- 2000)	6001 (0)	3	1-5	91.9	48.6 (13.1)	2556 (49.8)	1039 (40.6, 45.8)
6A13	Q6_3	Current marital status	2001	1049 (0)	2	1-6	100	47.1 (7.9)	438 (53.4)	188 (42.9, 49.5)
6A14	Q12_3	Current marital status	2006	1854 (0)	2	1-8	100	55.4 (11.7)	763 (52.6)	357 (46.8, 49)
6A15	Q14_3	Current marital status	2008	5114 (0)	1	1-5	88.5	55.1 (13.5)	2098 (55.7)	881 (42, 53.6)
6A16	Q17D_4	Current marital status	2009 (2007- 2010)	3513 (4)	1	1-6	99.5	58.5 (10.2)	1701 (52.1)	768 (45.1, 50.9)

**Table 6Bi.** Marriage history: divorce i. Summary of responses for TwinsUK participants in response to question: **6B1.** (Q8_9) Have you ever been divorced? **6B2.** (Q17D_9) In the past ten years did you get divorced/ separated? **6B3.** (Q11A_34) Have you ever been divorced? %Yes = % yes response, %F = % female responses, SD = standard deviation, N twin pairs = Number of twin pairs where both responded, %MZ = percentage of responding twin pairs who are mono-zygotic (identical) twins, %D = % of responding twin pairs considered discordant, %MZD = percentage of discordant twin pairs who are mono-zygotic.

	TwinsUK reference code	Question description	Response year (mean, range)	N responses (duplicate ids)	%Yes	%F	Age (mean, SD)	N twin pairs (%MZ)	Discordant twin pairs (%D, %MZD)
6B1	Q8_9	Ever been divorced	2001 (all)	4614 (0)	20.9	88.2	50.3 (12.9)	1774 (50.7)	542 (48.3)
6B2	Q17D_9	Got divorced separated past ten years	2009 (2007- 2010)	694 (0)	36.2	99	55.5 (11.4)	87 (62.1)	26 (57.7)
6B3	Q11A_34	Ever been divorced	2012 (2004- 2014)	5656 (2690)	27.6	88.9	57.3 (11.5)	1851 (56.8)	617 (33.3, 56.8)

**Table 6Bii.** Marriage history: divorce i. Summary of responses for TwinsUK participants in response to question: **6B4.** (Q11A_33) How many times have you been married? %F = % female responses, SD = standard deviation, N twin pairs = Number of twin pairs where both responded, %MZ = percentage of responding twin pairs who are mono-zygotic (identical) twins, %D = % of responding twin pairs considered discordant, %MZD = percentage of discordant twin pairs who are mono-zygotic.

	TwinsUK reference code	Question description	Response year (mean, range)	N responses (duplicate ids)	Population mean	SD	Range	%F	Age at response (mean, SD)	N twin pairs (%MZ)	Discordant twin pairs (%D, %MZD)
6B4	Q11A_33	Number times married	2011 (2004- 2014)	5561 (2661)	1.2	0.7	0-10	88.9	57.4 (11.4)	1794 (56.4)	487 (27.1, 56.4)

**Table 6C.** Age when first married. Summary of responses for TwinsUK participants in response to question: **6C1** (Q11A_32) ‘How old were you when you first married?’ %F = % female responses, SD = standard deviation, N twin pairs = Number of twin pairs where both responded, %MZ = percentage of responding twin pairs who are mono-zygotic (identical) twins, %D = % of responding twin pairs considered discordant, %MZD = percentage of discordant twin pairs who are mono-zygotic.

	TwinsUK reference code	Question description	Response year (mean, range)	N responses (duplicate ids)	Population mean	SD	Range	%F	Age at response (mean, SD)	N twin pairs (%MZ)	Discordant twin pairs (%D, %MZD)
6C1	Q11A_32	Age when first married	2010 (2004- 2014)	5587 (2684)	24.3	5.4	14-64	89	57.6 (11.3)	1816 (56.4)	415 (22.9, 56.4)

**Table 6D.** Ever been married. Summary of responses for TwinsUK participants in response to question: **6D1** (Q11A_29) ‘Have you ever been married?’ %Yes = % yes response, %F = % female responses, SD = standard deviation, N twin pairs = Number of twin pairs where both responded, %MZ = percentage of responding twin pairs who are mono-zygotic (identical) twins, %D = % of responding twin pairs considered discordant, %MZD = percentage of discordant twin pairs who are mono-zygotic.

	TwinsUK reference code	Question description	Response year (mean, range)	N responses (duplicate ids)	%Yes	%F	Age at response (mean, SD)	N twin pairs (%MZ)	Discordant twin pairs (%D, %MZD)
6D1	Q11A_29	Ever been married	2008 (2004-2014)	6220 (2906)	88.9	88.5	56.1 (13.2)	2131 (57.6)	231 (10.8, 59.3)

**Table 6E.** Length of relationship. Summary of responses for TwinsUK participants in response to question: **6E1** (PH0001335) ‘How long have you been together with your current partner? (Years’) %F = % female responses, SD = standard deviation, N twin pairs = Number of twin pairs where both responded, %MZ = percentage of responding twin pairs who are mono-zygotic (identical) twins, %D = % of responding twin pairs considered discordant, %MZD = percentage of discordant twin pairs who are mono-zygotic.

	TwinsUK reference code	Question description	Response year (mean, range)	N responses (duplicate ids)	Population mean	SD	Range	%F	Age at response (mean, SD)	N twin pairs (%MZ)	Discordant twin pairs (%D, %MZD)
6E1	PH0001335	Relationship length years	2013 (all)	2842 (0)	30.3	15.8	0-65	89	59.1 (12.8)	785 (60.3)	172 (21.9, 48.8)

### Limitations of data

Not all the participants here will have longitudinal and/or multi-omics data, and therefore the specific numbers available to interested parties will differ dependent on the research question.

## Data availability

### Underlying data

TwinsUK welcomes and encourages data to be shared within the scientific community to help assist and generate further scientific research, and in particular is interested in facilitating the use of our data by social scientists. To ensure the privacy of participants is maintained and to guard against misuse of the resource, data is accessible by managed access only. Researchers who would like access to the datasets which have been presented in this data note in addition to other TwinsUK data can follow the steps provided below:

1. Please use the TwinsUK Phenotype spreadsheet containing a list of all TwinsUK phenotypes to search for the specific phenotype required.

2. Please read through the TwinsUK Data Access Policy document which explains how to access the data and samples in addition to the cost. There are a limited number of grants available to waive any costs to any researcher whose research scope can be considered ‘social science’. 

3. Please send your Proposal Form to the TwinsUK Resource Executive Committee (TREC) for it to be considered. You will receive a notification about the conclusion of the review within three weeks of submitting and then be advised on the next step of the process.

The data and/or samples required including individual variables should be specified on the proposal form with an appropriate reason outlining the aims/hypothesis of the project for which the data is needed. If further information is required, it can be found on the TwinsUK website. If there are any other enquires linked to data access please email Collaborations and Data Access Manager, Victoria Vazquez ( victoria.vazquez@kcl.ac.uk).
