# Supplementary material for: Diverging destinies: ‘social’ data within the TwinsUK cohort
Source: Wellcome Open Res. 2022 Jan 24;7:19. [Version 1] doi: 10.12688/wellcomeopenres.17139.1 (PMC10558976; doi:10.12688/wellcomeopenres.17139.1)
Supplement: Peer review history

# Reviewer report: Reviewer response for version 1

**DOI:** 10.21956/wellcomeopenres.18932.r66883

**Published:** 2023-10-06

**Authors:** Margot P van de Weijer

**Affiliations:** 1 Psychiatry, Amsterdam University Medical Centres, Amsterdam, North Holland, The Netherlands

I believe this paper provides a useful summary of the social data present in the TwinsUK dataset, which will be helpful for (twin) researchers when considering to use TwinsUK data. I have two minor comments, both with respect to where I believe the paper would benefit from more details:

1. The first paragraph of the introduction very briefly mentions how twin data can be used as a sort of "natural experiment" when you have discordant twin pairs. I think the rationale behind this is clear for twin researchers, but I imagine quite vague for others not as familiar with twin research. It think it would benefit the paper if the authors would elaborate some more on how and why (discordant) twin data can be used.
2. The limitations section only mentions that not all participants will have longitudinal or omics data, but I imagine there might be some other limitations with respect to the data's representativeness?

Are sufficient details of methods and materials provided to allow replication by others?

Yes

Is the rationale for creating the dataset(s) clearly described?

Yes

Are the datasets clearly presented in a useable and accessible format?

Yes

Are the protocols appropriate and is the work technically sound?

Yes

Reviewer Expertise:

Behavior genetics, Gene-Environment Interplay

I confirm that I have read this submission and believe that I have an appropriate level of expertise to confirm that it is of an acceptable scientific standard.

# Reviewer report: Reviewer response for version 1

**DOI:** 10.21956/wellcomeopenres.18932.r64346

**Published:** 2023-08-23

**Authors:** Martin Diewald

**Affiliations:** 1 Bielefeld University, Bielefeld, Germany

This is a very helpful article to make the data more visible and accessible to researchers from different disciplines. The social information is overall parsimonious but contains several important pieces of information including, very welcome, longitudinal data. I have only a few recommendations for how the article could be improved, the first two being very easy to include:

1. Mention in the first paragraph that twin methodology is more than the basic ACE model but includes more sophisticated models to take into account gene-environment interaction and covariation, for which the relatively high number of cases is an important asset.
2. Mention that comparing MZ twins only is kind of a silver bullet for causal inference.
3. It would be helpful to have in addition information about age distribution (e.g., age brackets with respective n of cases). I know that there are some decisions to make how to cope with different measurement time points, but an aggregate information would be enough.
4. Some information about selectivity when including social information in addition to health would be interesting (representativeness of the data), but this is less urgent.

Are sufficient details of methods and materials provided to allow replication by others?

Partly

Is the rationale for creating the dataset(s) clearly described?

Yes

Are the datasets clearly presented in a useable and accessible format?

Yes

Are the protocols appropriate and is the work technically sound?

Yes

Reviewer Expertise:

social stratification, behavioural genetics, organizational inequalities, work-life interface, education and training

I confirm that I have read this submission and believe that I have an appropriate level of expertise to confirm that it is of an acceptable scientific standard.
